# 

**Published:** 1845-06

**Authors:** 


### CONTENTS

OF NO. VI.

---

### ORIGINAL COMMUNICATIONS.

### ESSAYS AND CASES.

Art. I.—A List of the Plants growing spontaneously in the vicinity of Quincy, Florida. By A. W. Chapman, M.D. 461

Art. II.—Lectures on Diseases of the Chest. By J. R. Buck, M.D., Lecturer on the Theory and Practice of Medicine in the Louisville Summer School of Medicine. - - 484

Art. III.—A Case in which the Skin of a Child born of white parents was so discolored as to suggest the idea of mixed blood. By John Stackhouse, M.D., of Patriot, Ia. - 491

### REVIEWS.

Art. IV.—A Treatise on Poisons in relation to Medical Jurisprudence, Physiology, and the Practice of Physic. By Robert Christison, M.D., F.R.S.E.; Professor of Materia Medica in the University of Edinburgh; Fellow of the Royal College of Physicians, &c.; Member of the American Philosophical Society, of the Royal Academy of Medicine of Paris, &c., &c., &c. First American, from the Fourth Edinburgh Edition. Philadelphia: Ed. Barrington & Geo. D. Haswell. 1845. 8vo. pp. 756. - 495

Art. V.—A Practical Treatise on the Diseases peculiar to Women, illustrated by Cases derived from Hospital and Private Practice. By Samuel Ashwell, M. D., Member of the Royal College of Physicians, London, and Obstetric Physician and Lecturer to Guy's Hospital. First complete American, from the last London edition. With notes by *Paul B. Goddard*, M.D., M.A.P.S., M.A.N.S., &c. Philadelphia: Lea & Blanchard. 1845. 8vo. pp. 520. - 501

Art. VI.—On the Anatomy and Diseases of the Urinary and Sexual Organs. Containing the Anatomy of the Bladder and of the Urethra, and the treatment of the Obstructions to which these passages are liable. By G. J. Guthrie, F.R.S., Surgeon to the Westminster Hospital, and to the Royal Westminster Ophthalmic Hospital, &c., &c. From the Third London Edition. Philadelphia: Lea & Blanchard. 1845. 8vo. pp. 150. - - - - - - - - 513

Art. VII.—Principles of Forensic Medicine. By William A. Guy, M. B., Cantab., Fellow of the Royal College of Physicians; Professor of Forensic Medicine, King's College, London; Physician to the King's College Hospital, &c., &c., &c. First American Edition. With Notes and Additions: by Charles A. Lee, M.D., Professor of Materia Medica and General Pathology in Geneva College, &c., &c. New York: Harper & Brothers. 1845. 8vo. pp. 711. - - - - - - - - - - - 519

SELECTIONS FROM AMERICAN AND FOREIGN JOURNALS

Senecio Aureus. - - - - - - - - - 521
Rheumatism. - - - - - - - - - - 522
Poisoning by Hydrocyanic Acid. - - - - - - 524
Tracheotomy. - - - - - - - - - - 525
Efficacy of Digitalis in a case of Pulmonary Hepatization. - - 526
Radical cure of Hydrocele. - - - - - - - - 526
Case of Twins. - - - - - - - - - 527
Galvanism in Uterine Hemorrhage. - - - - - - 527
Liquefaction and Solidification of Gases. - - - - - 528
Malpractice in a Case of Midwifery. - - - - - 529
Hernia. - - - - - - - - - - - 530
Opiates in Extensive Burns. - - - - - - - - 531
Poison of Snakes in Australia, - - - - - - - 531
Growing Potatoes by Galvanism. - - - - - - - 531
Death of Professor Daniell. - - - - - - - - 532
Foreign Bodies in the System. - - - - - - - 533
A Lecture on the Modern Improvements in Surgery. - - 534

ORIGINAL INTELLIGENCE

Medical Society of Tennessee, - - - - - - - 545
Esquirol on Insanity. - - - - - - - - - 546
A New Medical Journal. - - - - - - - - 547
New Works. - - - - - - - - - - - 547
Dictionary of Practical Medicine. - - - - - - - 548
Worcester Lunatic Asylum. - - - - - - - - 548

